# Detection of a new pyrethroid resistance mutation (V410L) in the sodium channel of *Aedes aegypti*: a potential challenge for mosquito control

**DOI:** 10.1038/srep46549

**Published:** 2017-04-19

**Authors:** Khalid Haddi, Hudson V. V. Tomé, Yuzhe Du, Wilson R. Valbon, Yoshiko Nomura, Gustavo F. Martins, Ke Dong, Eugênio E. Oliveira

**Affiliations:** 1 Departamento de Entomologia, Universidade Federal de Viçosa, Viçosa, MG 36570-900, Brasil; 2 Science Without Border Associate Researcher, Programa de Pós-Graduação em Entomologia, Universidade Federal de Viçosa, Viçosa, MG 36570-000, Brasil; 3 EAG Laboratories, 13709 Progress Blvd. #24 Suite S163, Alachua, FL, 32615 USA; 4 Department of Entomology, Genetics and Neuroscience Programs, Michigan State University, East Lansing, MI, USA; 5 Departamento de Biologia Geral, Universidade Federal de Viçosa, Viçosa, M G 36570-900, Brasil

## Abstract

The yellow fever mosquito, *Aedes aegypti*, particularly in Neotropical regions, is the principal vector of dengue, yellow fever, Zika and Chikungunya viruses. Pyrethroids remain one of the most used insecticides to control *Aedes* mosquitoes, despite the development of pyrethroid resistance in many mosquito populations worldwide. Here, we report a Brazilian strain of *A. aegypti* with high levels (approximately 100–60,000 fold) of resistance to both type I and type II pyrethroids. We detected two mutations (V410L and F1534C) in the sodium channel from this resistant strain. This study is the first report of the V410L mutation in mosquitoes. Alone or in combination with the F1534C mutation, the V410L mutation drastically reduced the sensitivity of mosquito sodium channels expressed in *Xenopus* oocytes to both type I and type II pyrethroids. The V410L mutation presents a serious challenge for the control of *A. aegypti* and will compromise the use of pyrethroids for the control of *A. aegypti* in Brazil; therefore, early monitoring of the frequency of the V410L mutation will be a key resistance management strategy to preserve the effectiveness of pyrethroid insecticides.

With vaccines unavailable for most of the arboviruses transmitted by mosquitoes, vector control has long been a key strategy to manage mosquito-associated diseases. The essential focus of this strategy is the elimination of breeding sites and the application of insecticides against both adults and larvae^1^,^2^,^3^,^4^,^5^. Our current mosquito control strategies have been the target of harsh condemnation^2^, mostly based on the spectacular increase in the global prevalence and incidence of dengue (DENV) and more recently, chikungunya (CHIKV) and Zika (ZIKV) arboviruses. For example, as of June 2016, 61 countries and territories report continuing Zika mosquito-borne transmission, with 47 experiencing a first outbreak of Zika virus^4^. In Brazil, more than 1600 cases of microcephaly and/or central nervous system malformation have been confirmed that are potentially associated with Zika virus infection^4^.

*Aedes aegypti* is the primary vector for Zika and dengue worldwide and also has high vectorial capacity for chikungunya and yellow fever arboviruses^2^,^4^,^5^. The prevention or reduction of transmission of these arboviruses is mostly dependent on the control of mosquitoes and limiting their contacts with humans. Among the various compounds (e.g., organophosphates and neonicotinoids for control of larvae and adults and insect growth regulators (IGR) and *Bacillus*-based compounds for control of larvae) recommended by the World Health Organization (WHO) in the effort to control mosquitoes, pyrethroid insecticides are one of most important because of their efficacy and safety^1^,^2^,^3^,^4^,^5^.

Pyrethroids exert their toxic effects by disrupting the functionalities of the voltage-gated sodium channels in the insect nervous system^6^,^7^,^8^,^9^. However, the development of pyrethroid resistance is the primary threat to the sustained use of pyrethroids for mosquito control^8^,^9^,^10^,^11^,^12^. A well-known mechanism of pyrethroid resistance, knockdown resistance (kdr), is caused by naturally occurring mutations in the voltage-gated sodium channel, which occur in a wide range of medically and agriculturally important arthropods, including disease vectors^7^,^10^,^13^,^14^,^15^. Several kdr mutations are recorded in the sodium channels of *A. aegypti* populations^10^,^16^,^17^,^18^, but only some of these mutations reduce the sodium channel sensitivity to pyrethroids^7^,^11^,^19^,^20^.

Thus, understanding the molecular bases of pyrethroid resistance in *A. aegypti* is crucial for early detection and monitoring of the resistance phenomenon, and to the development of appropriate strategies to delay the occurrence and further spread of resistance. Here, in two Brazilian strains of *A. aegypti*, we evaluated the susceptibility and then demonstrated the contributions of metabolic and site-target alteration mechanisms in limiting the actions of pyrethroid insecticides. For the first time in mosquitoes, we describe a valine to leucine substitution (V410L) in the transmembrane segment 6 of domain I that contributed to high levels of resistance to both types (i.e., I and II) of pyrethroids. The prevalence of this mutation represents a new challenging factor for the control of *A. aegypti*.

## Results

### Characterization of the levels of resistance to type I and type II pyrethroids in larvae

**Figure 1:**
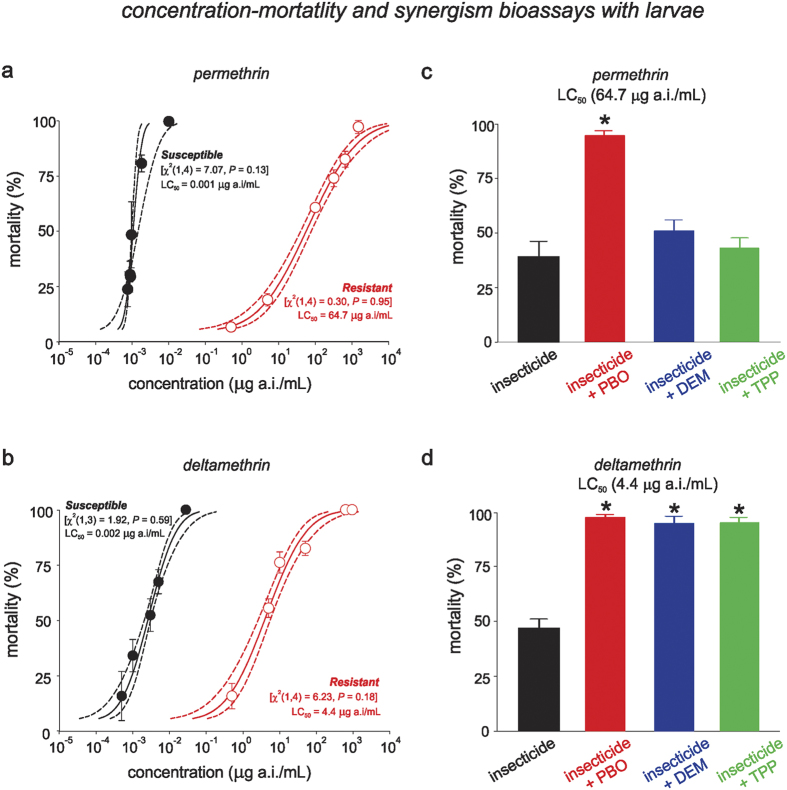
Toxicity (**a**,**b**) and comparative effects of synergists (**c**,**d**) on mortality caused by permethrin and deltamethrin to larvae (L4) of two Brazilian strains of *Aedes aegypti*. (**a**,**b**) The lines denote the lethal concentration (LC) values estimated based on concentration-mortality bioassays using probit analyses. Symbols show the averaged mortality for each insecticide concentration applied to each population of *A. aegypti*. The vertical bars represent the standard error of the average (SE). (**c**,**d**) The effects of the synergists piperonyl butoxide (PBO), diethyl maleate (DEM) and triphenyl phosphate (TPP) on the mortality of the resistant population caused by pyrethroids at the LC_50_ obtained for the resistant strain. *Asterisks* indicate significant differences in relation to the unsynergized insecticide (one-way ANOVA with Scheffe’s post hoc analysis, *P* < 0.05).

The analyses of the concentration-mortality parameters were based on the low χ2-values (<8.0) and high *P*-values (>0.05) obtained using the probit model (Table 1). The lethal toxicities of each pyrethroid to the larvae of the susceptible strain were similar, with LC_50_ values that varied from 0.001 to 0.003 μg of a.i./mL (Fig. 1). However, the resistance ratios (RR) to pyrethroids (compared to the LC_50_ for the larvae of the susceptible strain) was 59,830 (38,150–93,830)-fold to permethrin and 1,568 (772–3,186)-fold to deltamethrin (Table 1, Fig. 1A and B).

### High levels of pyrethroid resistance in larvae are the result of both enhanced metabolic detoxification and *kdr*-mutations

The bioassays with the three synergists (PBO, TPP, and DEM) revealed that the high levels of resistance to both pyrethroids (i.e., permethrin and deltamethrin) were partially based on enzymatic detoxification (Fig. 1C and D). The mortality to permethrin at the LC_50_ of the resistant population was significantly increased only by PBO (*df* = 3; *F* = 48.0; *P* < 0.001) (Fig. 1C), whilst all three synergists increased mortality to deltamethrin at the LC_50_ (*Deltamethrin: df* = 3; *F* = 128.3; P < 0.001) (Fig. 1D).

### High levels of pyrethroid resistance in adults are mainly due to *kdr*-mutations

**Figure 2:**
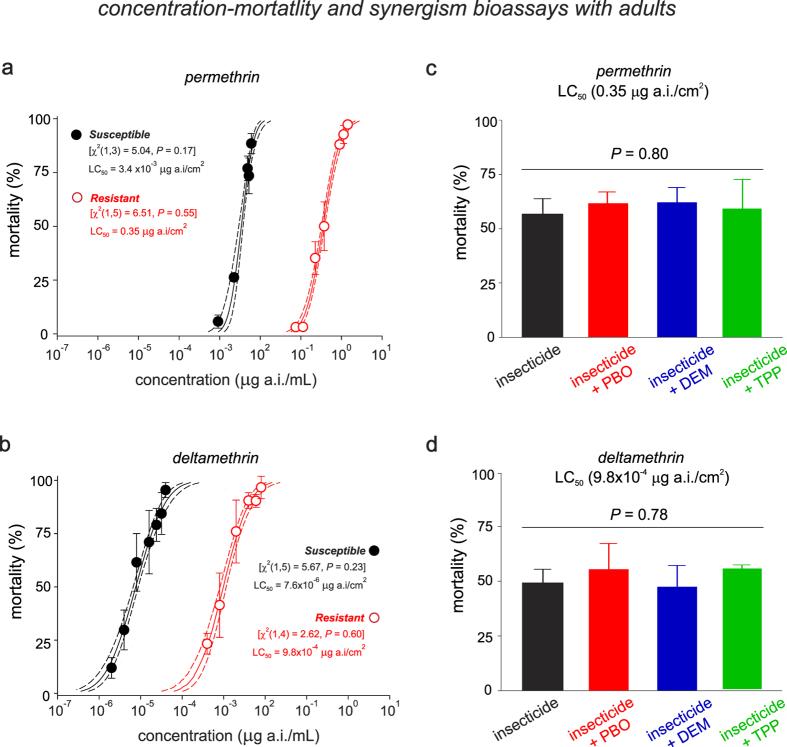
Toxicity (**a**,**b**) and comparative effects of synergists (**c**,**d**) on mortality caused by permethrin and deltamethrin to adults of two Brazilian strains of *Aedes aegypti*. (**a**,**b**) The lines denote the lethal concentration (LC) values estimated based on concentration-mortality bioassays using probit analyses. Symbols show the averaged mortality for each insecticide concentration applied to each population of *A. aegypti*. The vertical bars represent the standard error of the average (SE). (**c**,**d**) The effects of the synergists piperonyl butoxide (PBO), diethyl maleate (DEM) and triphenyl phosphate (TPP) on the mortality of the resistant population caused by pyrethroids at the LC_50_ obtained for the resistant strain. *Asterisks* indicate significant differences in relation to the unsynergized insecticide (one-way ANOVA with Scheffe’s post hoc analysis, *P* < 0.05).

For the results from the bioassays with adult mosquitoes, while the LC_50_ for permethrin in susceptible mosquitoes was 3.4 × 10^−3^ (3.0 × 10^−3^ − 3.8 × 10^−3^) μg a.i./cm^2^(Table 1, Fig. 2A), the LC_50_ for deltamethrin was 7.6 × 10^−6^ (6.6 × 10^−6^ − 8.8 × 10^−6^) μg a.i./cm^2^ (Table 1, Fig. 2B). Based on these LC_50_ measures, permethrin was 243.2 (182.7–323.8)-fold less toxic than deltamethrin. Compared to the susceptible strain, the resistant strain was 102.2 (73.7–141.7)-fold more resistant to permethrin and 129.0 (88.4–188.3)-fold more resistant to deltamethrin (Table 1, Fig. 2A and B).

In contrast to results for the larval assays, pre-treatment with three synergists, PBO, TPP and DEM, did not enhance the toxicities of permethrin nor deltamethrin (Fig. 2C and D) in adult mosquitoes (*Permethrin: F* = 0.36; *P* = 0.78. *Deltamethrin: F* = 0.33; *P* = 0.80).

### First record of V410L mutation (alone and together with F1534C mutation) in the sodium channel gene of *A. aegypti*

**Figure 3:**
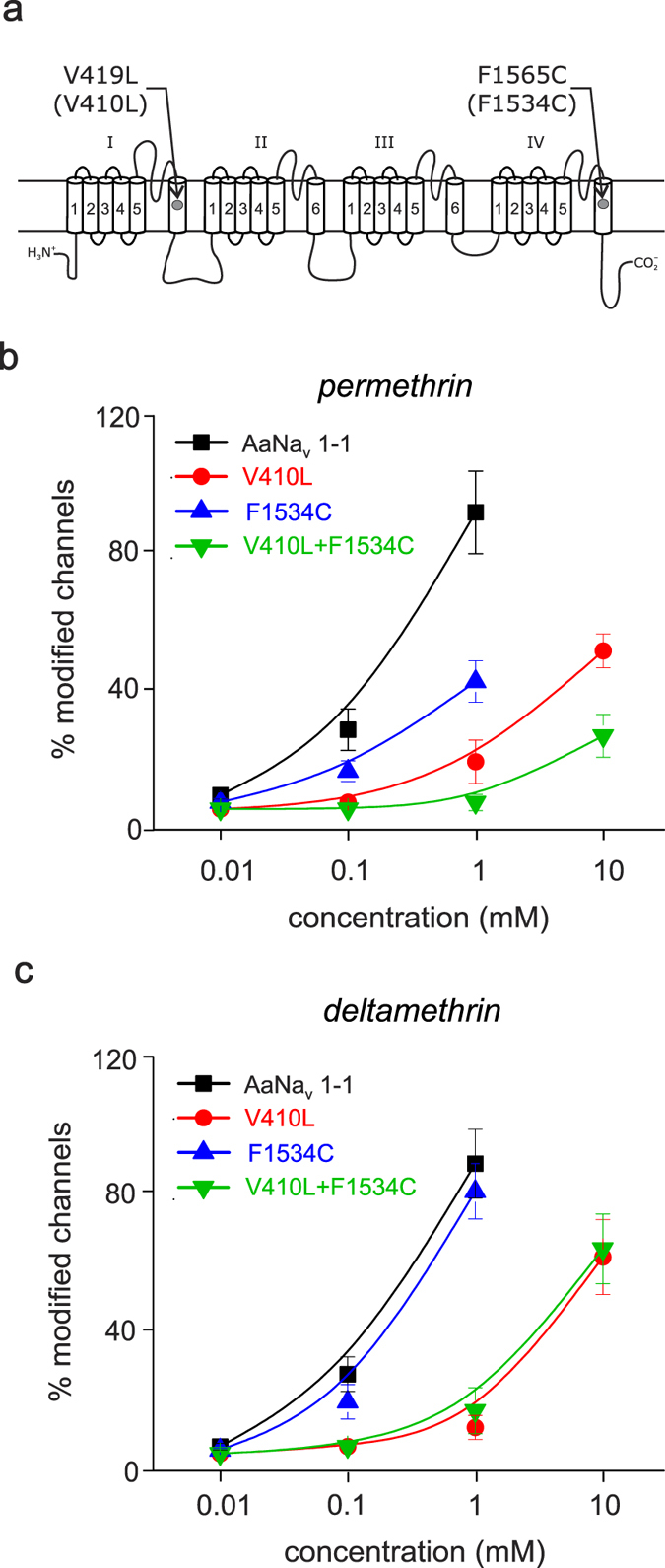
Pyrethroid resistance-associated amino acid substitutions in *A. aegypti* sodium channels. (**a**) Schematic representation of the mosquito sodium channel protein (AaNa_v_1-1) indicating two knockdown resistance mutations in IS6 and IVS6. Sodium channels are large transmembrane proteins with four homologous repeats (I–IV), each having six transmembrane segments (S1–S6). Residue positions correspond to the cockroach BgNa_v_ protein (GenBank number: U73583). Dose-response curves of permethrin (**b**) and deltamethrin (**c**) on BgNav1-1a, V410L, F1534C and V410L + F1534C cockroach sodium channels. The method for determining the percentage of modified channels is described in the Materials & Methods section.

To identify possible genetic modifications responsible for the high levels of pyrethroid resistance, the complete coding region of the sodium channel from the pyrethroid-resistant (Accession number: KY747530) and pyrethroid-susceptible (Accession number: KY747529) strains was cloned and sequenced. Sequence comparison revealed eight nucleotide alterations between pyrethroid-resistant and pyrethroid-susceptible populations of which only two resulted in amino acid substitutions (Fig. 3A). One substitution was at position 410 in the transmembrane segment 6 of domain I (IS6) in which a valine was substituted by a leucine (V410L), and the other substitution was at position 1534 in the transmembrane segment 6 of domain III (IIIS6) in which a phenyl-alanine was substituted by a cysteine (F1534C). The comparison of genomic DNA sequences of 25 individual mosquitoes from both susceptible and resistant populations of *A. aegypti* confirmed these amino acid substitutions. Our results showed that all the sequenced pyrethroid resistant mosquitoes had the F1534C mutation and 7 of the 10 also carried the V410L mutation. The allele frequencies were 100 and 50% for both mutations, respectively.

### V410L mutation confers AaNav1-1 channel resistance to both type I and type II pyrethroids

To assess the effects of the two mutations on pyrethroid resistance, we used the pyrethroid-sensitive mosquito sodium channel AaNa_v_1-1^20^ to generate mutant channels expressing either single (i.e., V410L or F1534C) or double mutations (i.e., V410L + F1534C). Consistent with previous findings^17^,^20^,^21^, the F1534C mutation significantly decreased the AaNav1-1 sensitivity to permethrin (Fig. 3B) but not to deltamethrin (Fig. 3C). Interestingly, the V410L mutation alone reduced the sensitivity of mosquito AaNav1-1 to both deltamethrin and permethrin by approximately 10-fold (Fig. 3B and C), and the V410L and F1534 double mutations further enhanced the resistance of AaNa_v_1-1 channels to permethrin compared with the two single mutations (Fig. 3B). These results indicate an additive effect of the two mutations on permethrin resistance.

### Screening of wild populations for the presence of kdr mutations

The sequencing of two fragments harboring the *kdr* mutations (V410L and F1534C) showed that none of the 11 field collected populations has the V410L mutation while the F1534C substitution was detected in five populations (Table 2). When present, the F1534C mutation was always heterozygous (Table 2).

## Discussion

In this study, we report on a Brazilian strain of *A. aegypti* with high levels of pyrethroid resistance conferred primarily by *kdr* (i.e., V410L and F1534C) mutations, especially in adult mosquitoes. The V410L mutation is reported for the first time in the sodium channels of mosquitoes, and by identifying the valine to leucine substitution at a corresponding position in a mosquito (i.e., AaNa_v_1-1) sodium channel, we demonstrated that this amino acid substitution reduced the sensitivity of these channels to both type I (i.e., permethrin) and type II pyrethroids (i.e., deltamethrin).

Vector control has long been a key strategy in managing mosquito-borne human diseases, and the application of pyrethroid insecticides continues to be one of most important components in this effort^1^,^2^,^3^,^4^,^5^. However, the development of pyrethroid resistance is a major threat to the sustained use of pyrethroids in vector control. Historically, resistance has developed in Brazilian populations of disease-vector mosquitoes to a wide range of insecticides, including pyrethroids^16^,^22^,^23^,^24^,^25^.

Although more than 50 sodium channel mutations are associated with pyrethroid resistance in diverse arthropod pests, not all of the mutations cause resistance by reducing the docking of the pyrethroid at the sodium channels^7^,^11^,^19^,^20^. Such was likely the case for the V410L mutation because the amino acid substitution was not located in one of the two recently described pyrethroid receptor sites^11^,^19^,^20^,^26^. One potential alternative explanation is that some *kdr* mutations reduce pyrethroid effects by altering the gating properties of the channel without inhibiting molecule docking. This altered gating could contribute to resistance because pyrethroids prefer binding to sodium channels in an open state, and *kdr* mutations that deter gating transitions to the open state would counteract the pyrethroid effects^9^. For example, altered kinetics of channel gating and reduced channel sensitivity to pyrethroids are reported when an aspartic acid (D) is replaced by a lysine (K) at position 802 in IIS1 in a cockroach (BgNav) splicing variant sodium channel, in addition to when valine to leucine or isoleucine substitutions occur at the 410 corresponding position in a cockroach sodium channel (BgNav1-1a)^27^,^28^.

More than one *kdr* mutation associated with insecticide resistance are frequently found in insects^7^,^13^, including in *A. aegypti*^10^,^16^,^17^,^18^. The occurrence of both V410L and F1534C mutations might explain the very high levels of resistance found in this study, indicating that monitoring strategies should focus on the screening of more than one sodium channel region. For example, the allele frequencies for the F1534C mutation were consistent with the wide distribution of F1534C previously reported in Brazil^25^, which would support the mosquito control programs that integrate the rotations between type I and type II pyrethroid products as a control strategy. This strategy could be successful because the F1534C mutation is confirmed to reduce the sensitivity of *A. aegypti* AaNav1-1 channels to type I (e.g., permethrin) but not to type II (e.g., deltamethrin) pyrethroids^17^,^21^. However, the very high levels of pyrethroid insensitivity conferred by the V410L mutation alone or in combination with F1534C could make pyrethroids not only largely ineffective in Brazilian regions in which *A. aegypti* have these mutations in combination but could also make the situation worse by increasing the pressure to select resistant individuals.

The screening of field populations from the Brazilian Pernambuco state revealed the absence of the mutation V410L which may indicate that this mutation is not widely spread in natural populations. Furthermore, half of the same samples were found to harbor the widely spread F1534C substitution, which helps to explain the resistance mechanisms for these mosquitoes populations^29^. The pyrethroids resistance in these populations was reported to be correlated not only with different *kdr* mutations (e.g., the I1011M) but also with detoxification mechanisms^29^, indicating clearly the contribution of various mechanisms to the reported cases of pyrethroid resistance in Brazil.

In the present investigation, for instance, we demonstrated the contribution of various mechanisms to the high level of pyrethroid resistance in the resistant strain, especially in larvae. The pre-exposure of larvae from the resistant strain to the synergist PBO increased the mortality caused by both deltamethrin or permethrin alone, which indicate the involvement of the cytochrome P450-dependent monooxygenases^30^,^31^,^32^,^33^ and other targets (e.g., esterases) secondarily affected by this synergist^32^,^34^. Furthermore, based on our results from the synergism bioassays with TPP and DEM and as previously demonstrated in insects^35^, the esterases and also glutathione-S-transferases do also contribute to the high resistance level to deltamethrin in larvae of the resistant strain. However, the high resistance level recorded for adults of the resistant strain is mainly due to *kdr*-mutations as the pre-treatment of the three synergists did not increase the toxicities of permethrin and deltamethrin in adult mosquitoes. It is clear that there are enormous gaps in our knowledge of insect enzymes.

Thus, further investigations aiming to identify the distribution of the V410L mutation in Brazilian populations of *A. aegypti* and on the correlation of the mutation with pyrethroid resistance in field populations are warranted. Additionally, the effects of this mutation on the ability of mosquitoes to transmit human arboviruses also require investigation.

## Materials and methods

This study did not use any live vertebrates and followed the laws and ethical guidelines of Brazil. Furthermore, owing to the nature of specimens used (i.e. this study did not involve any endangered or protected species) and as all mosquitoes used were reared at the insectary of the Department of General Biology, Federal University of Viçosa (Viçosa, MG, Brazil), no additional permissions or approvals were required.

### Mosquito strains used in the concentration mortality bioassays

The two strains used in the concentration-mortality bioassays were the pyrethroid-susceptible PPCampos population (Campos dos Goytacazes, Rio de Janeiro State, Brazil) and the pyrethroid-resistant F2 Oiapoque population (Rio de Janeiro, Rio de Janeiro State, Brazil). Mosquito strains were maintained in the insectary of the Department of General Biology, Federal University of Viçosa (Viçosa, MG, Brazil), under controlled conditions of temperature (25 ± 3 °C), relative humidity (60 ± 2%), and photoperiod (12:12 L:D), as previously described in Marriel *et al*.^36^.

### Characterization of mosquito sensitivity to pyrethroids

We conducted concentration mortality bioassays using larvae and adults. The concentration-mortality bioassays using mosquito were conducted based on the protocol recommended by WHO^37^. In brief, five repetitions of 25 early fourth instar larvae (L4) were put in glass jars (200 mL) with 100 mL of insecticide solutions at various concentrations, which caused 5–95% mortality. The control group was maintained in 100 ml of distilled water. Mortality was recorded 24 h after treatment. At least 6 different concentrations were used for each insecticide. We used commercial formulations of the pyrethroids permethrin (25 g a.i./L, BASF Brasil, São Paulo - SP, Brazil) and deltamethrin (25 g a.i./L, Bayer Crop Science, São Paulo - SP, Brazil).

For the concentration-mortality bioassays with adults, glass-vial based bioassays were carried out. The inner wall of 200 mL glass vials was impregnated with 2 mL of insecticide solution at various concentrations and left to dry by rotation for 2 h. At least five different concentrations were used for each insecticide. 25 to 30 one- to two-day old starved females were exposed in each vial and four vials were used for each insecticide concentration. Mortality was assessed after 24 h exposure.

### Bioassays using synergists that inhibit detoxification enzymes

The contribution of enzymatic detoxification was quantified by measuring the mortality of resistant L4 as well as adults that were previously exposed to the following synergists: triphenyl phosphate (TPP; an esterase inhibitor), diethyl maleate (DEM; a glutathione S-transferase inhibitor), and piperonyl butoxide (PBO; an inhibitor of cytochrome P450-dependent monooxygenases and esterases). Preliminary tests with varying concentrations of synergists were carried out to determine the maximum sublethal concentrations for adult and larvae mosquitoes. Subsequently, these maximum sublethal concentrations were used in the synergism tests were of 1.5%, 2.5 and 2% for PBO, DEM, and TPP, respectively.

For the bioassays using larvae, the synergists were dissolved in acetone, and 1 mL of the synergist solutions was added to 99 mL of distilled water. Twenty-five early L4 were transferred to glass jars and remained in contact with a synergist for 1 h before being exposed to an insecticide solution (at the corresponding lethal concentration capable of killing 50% of tested larvae - LC_50_). The L4 exposed to synergists were then transferred to other glass jars containing 100 mL of insecticide solutions. Two control groups were used, one with only 100 mL of distilled water and the other with the addition of 1 ml acetone into to 99 mL of distilled water. Mortality was recorded 24 h after insecticide treatment.

For adults, the bioassay was similar to the one for the concentration mortality. The inner wall of 200 mL glass vials was impregnated, at various concentrations, with 2 mL of insecticide solution containing acetone dissolved synergists. As controls, we used vials impregnated with water alone, with water and acetone. Mortality was recorded 24 h after insecticide treatment.

### Cloning and sequencing of cDNA and genomic DNA of the sodium channel gene from *A. aegypti*

Total RNA from pools of 15-20 adult mosquitoes of each population was extracted using a PureLink^®^ RNA Mini Kit according to the manufacturer’s protocol (Life Technologies, Carlsbad, California, USA). Subsequently, first-strand cDNA was synthesized from total RNA using SuperScript III reverse transcriptase (Invitrogen, Carlsbad, California, USA). PCRs were performed with specific primer pairs designed based on the *A. aegypti* sequence available in GenBank (accession number AY663385) (Supplementary Table S1). PCR reactions (20 μL) consisted of 1.2 μL of template cDNA, 0.75 μL of each primer (10 μM), 12 μl of GreenTaq (Fermentas, Waltham, Massachusetts, USA) and 6 μl of sterile distilled water. PCR cycling conditions were as follow: 94 °C for 2 min; 35 cycles at 94 °C for 45 s, at 58 °C for 60 s, and at 72 °C for 120 s; and 72 °C for 7 min. The amplified fragments of the expected size were purified using Wizard SV gel and PCR Cleanup System from Promega (Madison, Wisconsin, USA) and then cloned using Stellar^TM^ Competent Cells from Clonetech (Mountain View, California, USA) according to the manufacturer’s instructions. Five to six DNA clones for each segment were sent for sequencing at Macrogen facilities (Macrogen Inc., Seoul, South Korea).

To confirm the amino acid substitution mutations, genomic DNA was extracted separately from 10 insects. Primers flanking the potential mutation sites (Supplementary Table S1) were designed. PCRs were performed as follow: 94 °C for 2 min; 35 cycles at 94 °C for 40 s, at 58 °C for 45 s, and at 72 °C for 75 s; and 72 °C for 10 min. PCR products were directly sequenced (using internal primers) as described above.

### Functional characterization of the sensitivity of the AaNav1-1 and mutant mosquito sodium channels to pyrethroids in *Xenopus* oocytes

To introduce V410L, F1534C and V410L + F1534C mutations into a pyrethroid-sensitive mosquito sodium channel (AaNav1-1), site-directed mutagenesis was performed with Polymerase Chain Reaction (PCR) using Pfu Turbo DNA polymerase (Stratagene, La Jolla, CA, USA). All mutagenesis results were confirmed by DNA sequencing. Procedures for oocytes preparation and cRNA injection were identical to those described previously^38^. For robust expression of AaNav1-1 sodium channels, cRNA was co-injected into oocytes with cRNA encoding the *Drosophila melanogaster* tipE auxiliary subunit (1:1 ratio), which enhances the expression of insect sodium channels in oocytes^39^,^40^.

To quantify the effects of type I (i.e., permethrin) and type II (i.e., deltamethrin) pyrethroids, a two-electrode voltage clamp was used to measure pyrethroid-induced tail currents. Methods and data analysis for the two-electrode voltage clamp recording of sodium currents and measurement of tail currents induced by pyrethroids were identical to those previously described^38^,^41^. Briefly, the pyrethroid-induced tail current was recorded during a 100-pulse train of 5-ms step depolarizations from −120 to 0 mV with 5-ms interpulse intervals^42^. The percentage of channels modified by pyrethroids was calculated as follows: M = {[I_tail_/(E_h_ − E_Na_)]/[I_Na_/(E_t_ − E_Na_)]} × 100^41^, where I_tail_ is the maximal tail current amplitude, E_h_ is the potential to which the membrane is repolarized, E_Na_ is the reversal potential for sodium current determined from the current–voltage curve, I_Na_ is the amplitude of the peak current during depolarization before pyrethroid exposure, and E_t_ is the potential of step depolarization.

All experiments were performed at room temperature. Sodium currents were measured with an OC725C oocyte clamp (Warner Instruments, Hamden, CT) and a Digidata 1440 A interface (Axon Instruments Inc., Foster City, CA), and pCLAMP 10.2 software (Axon Instruments Inc.) was used for data acquisition and analysis. Deltamethrin and permethrin were kindly provided by Dr. Bhupinder Khambay (Rothamsted Research Institute, Hertfordshire, UK) and Dr. Ralf Nauen (Bayer CropScience AG, Monheim, Germany). The purities of different pyrethroids ranged from 99.3% to 99.8%. Stock solutions of pyrethroids (100 mM) were dissolved in dimethyl sulfoxide (DMSO). The working concentration was prepared in ND96 recording solution immediately before the experiments. The concentration of DMSO in the final solution was <0.5%, which had no effect on the function of sodium channels in the experiments.

### Screening of wild populations for the presence of kdr mutations

DNA samples of eleven field collected populations from Pernambuco state in the Brazilian Northeast region were generously obtained from the Departamento de Entomologia/CPqAM/Fiocruz-PE (Supplementary Table S2). The populations were initially established from eggs collected in “oviposition traps” in various municipalities of Pernambuco state. Their susceptibility to the pyrethroid type II cypermethrin were previously assessed and the resistance levels ranged from 7.1 to 222.6-fold^29^. We used the specific primers flanking the two mutation sites (Supplementary Table S1) under the same described PCRs conditions and PCR products were directly sequenced (using internal primers) as described above.

### Statistical Analyses

Concentration–mortality data were subjected to probit analysis (PROC PROBIT; SAS Institute 2008), and 95% CIs for resistance ratios (RRs) were estimated as reported by Robertson *et al*.^43^ and considered significant when they did not include the value 1. The synergism results and those for the sodium channel sensitivity were subjected to one-way analysis of variance (one-way ANOVA) and to Scheffe’s post hoc analysis test (*P* < 0.05) to isolate the different treatments, using SigmaPlot 12.0 (Systat Software, San Jose, CA, USA).

### Ethical approval

All applicable international, national, and institutional guidelines for the care and use of animals were considered in the present investigation.

## Additional Information

**How to cite this article**: Haddi, K. *et al*. Detection of a new pyrethroid resistance mutation (V410L) in the sodium channel of Aedes aegypti: a potential challenge for mosquito control. *Sci. Rep.* **7**, 46549; doi: 10.1038/srep46549 (2017).

**Publisher's note:** Springer Nature remains neutral with regard to jurisdictional claims in published maps and institutional affiliations.

## Supplementary Material

Supplementary Information

## Figures and Tables

**Table 1:** Relative toxicity of type I and type II pyrethroids to larvae and adults of two Brazilian strains of *Aedes aegypti*.

Insect phase	Insecticide	Population	*n*	Slope ± S.E	LC_50_ (95% CI) larvae (μg a.i./mL) adults (μg a.i./cm^2^)	*χ2*	*P-*values^a^	*RR*^b^ LC_50_ (95% CI)^c^
Larvae (L4)	Permethrin *(type I pyrethroid)*	susceptible	600	16.2 ± 5.05	1.1 × 10^−3^ (9.8 × 10^−4^ –1.5 × 10^−3^)	7.07	0.13	—
		resistant	500	1.7 ± 0.15	64.7 (46.31–89.54)	0.30	0.95	59830.1 (38150.1–93830.4)
	Deltamethrin *(type II pyrethroid)*	susceptible	420	4.4 ± 0.56	2.7 × 10^−3^ (2.2 × 10^−3^ –3.3 × 10^−3^)	1.92	0.59	—
		resistant	420	0.7 ± 0.13	4.4 (2.94–6.18)	6.23	0.18	1568.0 (772.6–3186.2)
Adults	Permethrin *(type I pyrethroid)*	susceptible	591	2.6 ± 0.28	4.3 × 10^−3^ (4.0 × 10^−3^ –4.5 × 10^−3^)	5.04	0.17	—
		resistant	809	4.0 ± 0.27	0.35 (0.32–0.38)	6.51	0.55	102.2 (73.7–141.7)
	Deltamethrin *(type II pyrethroid)*	susceptible	671	5.3 ± 0.39	7.6 × 10^−6^ (6.6 × 10^−6^ –8.8 × 10^−6^)	5.67	0.23	—
		resistant	562	1.8 ± 0.13	9.8 × 10^−4^ (8.2 × 10^−4^ –1.2 × 10^−3^)	2.62	0.60	128.9 (88.4–188.3)

^a^Probability values.

^b^Resistance ratio (LC_50_ of resistant population/LC_50_ of susceptible population).

^c^When the 95% CI of RR included 1.0, the RRs were not significantly different (Robertson and Preisler, 1992).

**Table 2:** Frequencies of two *kdr* mutations (V410L and F1534C) in 11 Brazilian populations of *Aedes aegypti* sampled from Pernambuco state and reported resistant to type II pyrethroid.

Strains	*kdr* mutations	
	V410L	F1534C
Cedro	GTA	TGC
Recife	GTA	TGC
Santa Cruz do Capeberibe	GTA	TGC
São José do Egito	GTA	TGC
Afogados da Ingazeira	GTA	TTC
Serra Talhada	GTA	TTC
Itaíba	GTA	TGC
Arcoverde	GTA	TTC
Gloria do Goitá	GTA	TTC

(V: GTA; L: TTA; F: TTC; C: TGC).
