# Supplementary material for: Detection of a new pyrethroid resistance mutation (V410L) in the sodium channel of Aedes aegypti: a potential challenge for mosquito control
Source: Sci Rep. 2017 Apr 19;7:46549. doi: 10.1038/srep46549 (PMC5396194; doi:10.1038/srep46549)
Supplement: Supplementary Information [file srep46549-s1.pdf]

Supplementary Information

Detection of V410L mutation in sodium channel gene of *Aedes aegypti*: A new challenging factor for mosquito control

Khalid Haddi[a,b*], Hudson V.V. Tomé[a,c], Yuzhe Du[d], Wilson R. Valbon[a], Yoshiko Nomura[d], Gustavo F. Martins[e], Ke Dong[d], Eugênio E. Oliveira[a*]

[a] *Departamento de Entomologia, Universidade Federal de Viçosa, Viçosa, MG 36570-900, Brasil*

[b] *Science Without Border Associate Researcher, Programa de Pós-Graduação em Entomologia, Universidade Federal de Viçosa, Viçosa, MG 36570-000, Brasil*

[c] EAG Laboratories, 13709 Progress Blvd. #24 Suite S163, Alachua, FL, USA 32615

[d] *Department of Entomology, Genetics and Neuroscience Programs, Michigan State University, East Lansing, MI, USA*

[e] *Departamento de Biologia Geral, Universidade Federal de Viçosa, Viçosa, M G 36570-900, Brasil*

Supplementary Table S1. Primer sets used to sequence the voltage-gated sodium channel gene of *Aedes aegypti* and to analyze the mutations V410L and F1534C.

**Primer name and sequence**	**PCR amplification region**
AaAe F1: 5’-CATTGTTGGCCATATAGACAATG-3’<br>AaAe R1: 5’-ATCGACATCTTCTCCTTGTTG-3’	1 to 1536
AaAe F2: 5’-CGTACGACGAACTCCAGAAG-3’<br>AaAe R2: 5’-TAGCTCTACTATGCCGACCA-3’	1282 to 2167
AaAe F3: 5’- CAACGACAATCCTTTCATCG-3’<br>AaAe R3: 5’- CCATCGCACTCATCGTCTA-3’	2054 to 3647
AaAe F4: 5’-CAAGGACGAAAGCCACAAAG -3’<br>AaAe R4: 5’- GAACGACCCGAAGATGATG -3’	3536 to 4565
AaAe F5: 5’-GGCAAGTATTTTAAGTGCGT-3’<br>AaAe R5: 5’- CGTCCTCGTTGATGATACC-3’	4296 to 5326
AaAe F6: 5’-TACAATTTCAAGACGTTCGG-3’<br>AaAe R6: 5’-GCGAGGCCTGGCTCAGACATCCGC-3’	5245 to 6402
**Mutation V410L**<br>Ae410F1: 5’-TTACGATCAGCTGGACCGTG-3’<br>Ae410F2: 5’-ATCAGCTGGACCGTGGCA-3’<br>Ae410R1: 5’-TTCCTCGGCGGCCTCTTC-3’	1185 to 1334
**Mutation F1534L**<br>Ae1534F1: 5’-CGCGAGACCAACATCTACAT-3’<br>Ae1534R2: 5’-CTTTCAGCGGCTTCTTCGAG-3’<br>Ae1534R1: 5’-AGGCCGTGGAATAGCTTTC-3’	4546 to 4746

Supplementary Table S2.: Sampling sites, coordinates and collections dates of the *Aedes aegyptii* populations used in this study

Location	State	Coordinates	Collection date
Cedro	Pernambuco	7.7198° S, 39.2380° W	2010
Recife	Pernambuco	8.0476° S, 34.8770° W	2011
Agrestina	Pernambuco	8.4555° S, 35.9445° W	2010
Santa Cruz do Capibaribe	Pernambuco	7.9484° S, 36.2064° W	2010
San José do Egito	Pernambuco	7.4687° S, 37.2747° W	2010
Salgueiro	Pernambuco	8.0725° S, 39.1268° W	2010
Afogados da Ingazeira	Pernambuco	7.7480° S, 37.6347° W	2010
Serra Talhada	Pernambuco	7.9822° S, 38.2894° W	2012
Itaíba	Pernambuco	8.9470° S, 37.4212° W	2011
Arcoverde	Pernambuco	8.4177° S, 37.0585° W	2012
Gloria do Goitá	Pernambuco	7.9996° S, 35.2947° W	2010
